# Explainable AI in Fintech Risk Management

**DOI:** 10.3389/frai.2020.00026

**Published:** 2020-04-24

**Authors:** Niklas Bussmann, Paolo Giudici, Dimitri Marinelli, Jochen Papenbrock

**Affiliations:** ^1^FIRAMIS, Frankfurt, Germany; ^2^Department of Economics and Management, University of Pavia, Pavia, Italy; ^3^Fintech Laboratory, Department of Economics and Management, University of Pavia, Pavia, Italy

**Keywords:** credit risk management, explainable AI, financial technologies, peer to peer lending, logistic regression, predictive models

## Abstract

The paper proposes an explainable AI model that can be used in fintech risk management and, in particular, in measuring the risks that arise when credit is borrowed employing peer to peer lending platforms. The model employs Shapley values, so that AI predictions are interpreted according to the underlying explanatory variables. The empirical analysis of 15,000 small and medium companies asking for peer to peer lending credit reveals that both risky and not risky borrowers can be grouped according to a set of similar financial characteristics, which can be employed to explain and understand their credit score and, therefore, to predict their future behavior.

## 1. Introduction

Black box Artificial Intelligence (AI) is not suitable in regulated financial services. To overcome this problem, Explainable AI models, which provide details or reasons to make the functioning of AI clear or easy to understand, are necessary.

To develop such models, we first need to understand what “Explainable” means. During this year, some important benchmark definitions have been provided, at the institutional level. We report some of them, in the context of the European Union.

For example, the Bank of England (Joseph, 2019) states that “Explainability means that an interested stakeholder can comprehend the main drivers of a model-driven decision.” The Financial Stability Board (FSB, 2017) suggests that “lack of interpretability and auditability of AI and ML methods could become a macro-level risk.” Finally, the UK Financial Conduct Authority (Croxson et al., 2019) establishes that “In some cases, the law itself may dictate a degree of explainability.”

The European GDPR (EU, 2016) regulation states that “the existence of automated decision-making, should carry meaningful information about the logic involved, as well as the significance and the envisaged consequences of such processing for the data subject.” Under the GDPR regulation, the data subject is therefore, under certain circumstances, entitled to receive meaningful information about the logic of automated decision-making.

Finally, the European Commission High-Level Expert Group on AI presented the Ethics Guidelines for Trustworthy Artificial Intelligence in April 2019. Such guidelines put forward a set of seven key requirements that AI systems should meet in order to be deemed trustworthy. Among them three related to XAI, and are the following.

- Human agency and oversight: decisions must be informed, and there must be a human-in-the-loop oversight.
- Transparency: AI systems and their decisions should be explained in a manner adapted to the concerned stakeholder. Humans need to be aware that they are interacting with an AI system.
- Accountability: AI systems should develop mechanisms for responsibility and accountability, auditability, assessment of algorithms, data and design processes.

Following the need to explain AI models, stated by legislators and regulators of different countries, many established and startup companies have started to embrace Explainable AI (XAI) models.

From a mathematical viewpoint, it is well-known that, while “simpler” statistical learning models, such as linear and logistic regression models, provide a high interpretability but, possibly, a limited predictive accuracy, “more complex” machine learning models, such as neural networks and tree models provide a high predictive accuracy at the expense of a limited interpretability.

To solve this trade-off, we propose to boost machine learning models, that are highly accurate, with a novel methodology, that can explain their predictive output. Our proposed methodology acts in the post-processing phase of the analysis, rather than in the preprocessing part. It is agnostic (technologically neutral) as it is applied to the predictive output, regardless of which model generated it: a linear regression, a classification tree or a neural network model.

More precisely, our proposed methodology is based on Shapley values (see Lundberg and Lee, 2017 and references therein). We consider a relevant application of AI in financial technology: peer to peer lending.

We employ Shapley values to predict the credit risk of a large sample of small and medium enterprises which apply for credit to a peer to peer lending platform. The obtained empirical evidence shows that, while improving the predictive accuracy with respect to a standard logistic regression model, we maintain and, possibly, improve, the interpretability (explainability) of the results.

In other words, our results confirm the validity of this approach in discriminating between defaulted and sound institutions, and it shows the power of explainable AI in both prediction accuracy and in the interpretation of the results.

The rest of the paper is organized as follows: section 2 introduces the proposed methodology. Section 3 shows the results of the analysis in the credit risk context. Section 4 concludes.

## 2. Methodology

### 2.1. Credit Risk in Peer to Peer Lending

Credit risk models are useful tools for modeling and predicting individual firm default. Such models are usually grounded on regression techniques or machine learning approaches often employed for financial analysis and decision-making tasks.

Consider *N* firms having observation regarding *T* different variables (usually balance-sheet measures or financial ratios). For each institution *n* define a variable γ_*n*_ to indicate whether such institution has defaulted on its loans or not, i.e., γ_*n*_ = 1 if company defaults, γ_*n*_ = 0 otherwise. Credit risk models develop relationships between the explanatory variables embedded in *T* and the dependent variable γ.

The logistic regression model is one of the most widely used method for credit scoring. The model aims at classifying the dependent variable into two groups, characterized by different status (defaulted vs. active) by the following model:

(1) $$\begin{array}{l} ln\left(\frac{p_{n}}{1-p_{n}}\right)=\alpha +\overset{T}{\underset{t=1}{\sum }}\beta_{t}x_{nt} \end{array}$$

where *p*_*n*_ is the probability of default for institution *n*, **x**_*i*_ = (*x*_*i*, 1_, …, *x*_*i, T*_) is the *T*-dimensional vector of borrower specific explanatory variables, the parameter α is the model intercept while β_*t*_ is the *t*-th regression coefficient. It follows that the probability of default can be found as:

(2) $$\begin{array}{l} p_{n}=(1+exp\left(\alpha +\overset{T}{\underset{t=1}{\sum }}\beta_{t}x_{nt}\right){)}^{-1} \end{array}$$

### 2.2. Machine Learning of Credit Risk

Credit risk can be measured with very different Machine Learning (ML) models, able to extract non-linear relations among the financial information in the balance sheets. In a standard data science life cycle, models are chosen to optimize the predictive accuracy. In highly regulated sectors, like finance or medicine, models should be chosen balancing accuracy with explainability (Murdoch et al., 2019). We improve the choice selecting models based on their predictive accuracy, and employing *a posteriori* an explanations algorithm. This does not limit the choice of the best performing models.

To exemplify our approach we consider, without loss of generality, the XGBoost model, one of the most popular and fast algorithm (Chen and Guestrin, 2016), that implements gradient tree boosting learning models.

### 2.3. Learning Model Comparison

For evaluating the performance of each learning model, we employ, as a reference measure, the indicator γ ∈ {0, 1}, a binary variable which takes value one whenever the institutions has defaulted and value zero otherwise. For detecting default events represented in γ, we need a continuous measurement *p* ∈ [0, 1] to be turned into a binary prediction *B* assuming value one if *p* exceeds a specified threshold τ ∈ [0, 1] and value zero otherwise. The correspondence between the prediction *B* and the ideal leading indicator γ can then be summarized in a so-called confusion matrix. From the confusion matrix we can easy illustrate the performance capabilities of a binary classifier system. To this aim, we compute the receiver operating characteristic (ROC) curve and the corresponding area under the curve (AUC). The ROC curve plots the false positive rate (FPR) against the true positive rate (TPR), as follows:

(3) $$\begin{array}{l} FPR=\frac{FP}{FP+TN} \end{array}$$

(4) $$\begin{array}{l} TPR=\frac{TP}{TP+FN} \end{array}$$

The overall accuracy of each model can be computed as:

(5) $$\begin{array}{l} ACC=\frac{TP+TN}{TP+TN+FP+FN} \end{array}$$

and it characterizes the proportion of true results (both true positives and true negatives) among the total number of cases.

### 2.4. Explaining Model Predictions

We now explain how to exploit the information contained in the explanatory variables to localize and cluster the position of each individual (company) in the sample. This information, coupled with the predicted default probabilities, allows a very insightful explanation of the determinant of each individual's creditworthiness. In our specific context, information on the explanatory variables is derived from the financial statements of borrowing companies, collected in a vector **x**_*n*_ representing the financial composition of the balance sheet of institution *n*.

We propose calculate the Shapley value associated with each company. In this way we provide an agnostic tool that can interpret in a technologically neutral way the output from a highly accurate machine learning model. As suggested in Joseph (2019), the Shapley values of a model can be used as a tool to transfer predictive inferences into a linear space, opening a wide possibility of using the toolbox of econometrics, hypothesis testing, and network analysis.

We develop our Shapley approach using the SHAP (Lundberg and Lee, 2017) computational framework, which allows to express each single prediction as a sum of the contributions of the different explanatory variables.

More formally, the Shapley explanation model for each prediction $\phi \left(\hat{f}\left(x_{i}\right)\right)$ is obtained by an additive feature attribution method, which decomposes them as:

(6) $$\begin{array}{l} \phi \left(\hat{f}\left({\text{x}}_{i}\right)\right)=\phi_{0}+\overset{M}{\underset{k=1}{\sum }}\phi_{k}\left(x_{i}\right). \end{array}$$

where *M* is the number of available explanatory variables, ϕ ∈ ℝ^*M*^, ϕ_*k*_ ∈ ℝ. The local functions ϕ_*k*_(*x*_*i*_) are called Shapley values.

Indeed, Lundberg and Lee (2017) prove that the only additive feature attribution method that satisfies the properties of *local accuracy, missingness*, and *consistency* is obtained attributing to each feature *x*_*k*_, *k* = 1, …, *M*, a SHapley Additive exPlanation (SHAP) defined by

(7) $$\begin{array}{l} \phi_{k}\left(x_{i}\right)=\underset{x^{\prime }\subseteq C\left(x\right)\backslash x_{k}}{\sum }\frac{|x^{\prime }|!\left(M-|x^{\prime }|-1\right)!}{M!}\left[\hat{f}\left(x^{\prime }\cup x_{k}\right)-\hat{f}\left(x^{\prime }\right)\right], \end{array}$$

where $C\left(x\right)\backslash x_{k}$ is the set of all the possible models excluding variable *x*_*k*_ (with *m* = 1, …, *M*), |*x*′| denotes the number of variables included in model *x*′, *M* is the number of the available variables, $\hat{f}\left(x^{\prime }\cup x_{k}\right)$ and $\hat{f}\left(x^{\prime }\right)$ are the predictions associated with all the possible model configurations including variable *x*_*k*_ and excluding variable *x*_*k*_, respectively.

The quantity $\hat{f}\left(x^{\prime }\cup x_{k}\right)-\hat{f}\left(x^{\prime }\right)$ defines the contribution of variable *x*_*k*_ to each individual prediction.

## 3. Application

### 3.1. Data

We test our proposed model to data supplied by European External Credit Assessment Institution (ECAI) that specializes in credit scoring for P2P platforms focused on SME commercial lending. The data is described by Giudici et al. (2019a) to which we refer for further details. In summary, the analysis relies on a dataset composed of official financial information (balance-sheet variables) on 15,045 SMEs, mostly based in Southern Europe, for the year 2015. The information about the status (0 = active, 1 = defaulted) of each company 1 year later (2016) is also provided. Using this data, Giudici (2018); Ahelegbey et al. (2019), and Giudici et al. (2019a,b) have constructed logistic regression scoring models that aim at estimating the probability of default of each company, using the available financial data from the balance sheets and, in addition, network centrality measures that are obtained from similarity networks.

Here we aim to improve the predictive performance of the model and, for this purpose, we run an XGBoost tree algorithm (see e.g., Chen and Guestrin, 2016). To explain the results from the model, typically highly predictive, we employ Shapley values.

The proportion of defaulted companies within this dataset is 10.9%.

### 3.2. Results

We first split the data in a training set (80%) and a test set (20%).

**Figure 1:**
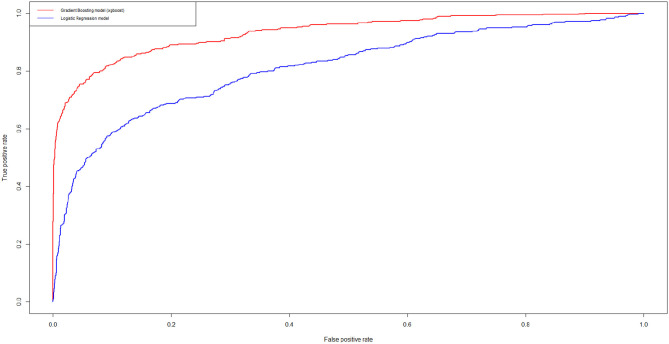
Receiver Operating Characteristic (ROC) curves for the logistic credit risk model and for the XGBoost model. In blue, we show the results related to the logistic models while in red we show the results related to the XGBoost model.

We then estimate the XGBoost model on the training set, apply the obtained model to the test set and compare it with the optimal logistic regression model. The ROC curves of the two models are contained in Figure 1 below.

From Figure 1 note that the XGBoost clearly improves predictive accuracy. Indeed the calculation of the AUROC of the two curves indicate an increase from 0.81 (best logistic regression model) to 0.93 (best XGBoost model).

We then calculate the Shapley values for the companies in the test set.

**Figure 2:**
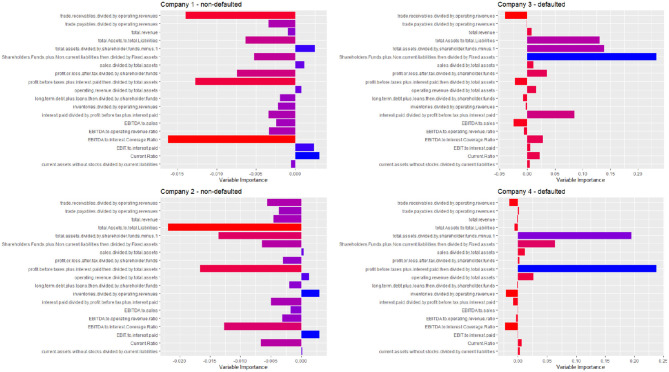
Contribution of each explanatory variable to the Shapley's decomposition of four predicted default probabilities, for two defaulted and two non-defaulted companies. A red color indicates a low variable importance, and a blue color a high variable importance.

To exemplify our results, Figure 2 we provide the interpretation of the estimated credit scoring of four companies: two that default and two that do not default.

Figure 2 clearly shows the advantage of our explainable model. It can indicate which variables contribute more to the prediction. Not only in general, as is typically done by feature selection models, but differently and specifically for each company in the test set. Note how the explanations are rather different (“personalized”) for each of the four considered companies.

## 4. Conclusions

The need to leverage the high predictive accuracy brought by sophisticated machine learning models, making them interpretable, has motivated us to introduce an agnostic, post-processing methodology, based on Shapley values. This allows to explain any single prediction in terms of the potential contribution of each explanatory variable.

Future research should include a better understanding of the predictions through clustering of the Shapley values. This can be achieved, for example, using correlation network models. A second direction would be to extend the approach developing model selection procedures based on Shapley values, which would require appropriate statistical testing. A last extension would be to develop a Shapley like measure that applies also to ordinal response variables.

Our research has important policy implications for policy makers and regulators who are in their attempt to protect the consumers of artificial intelligence services. While artificial intelligence effectively improve the convenience and accessibility of financial services, they also trigger new risks, and among them is the lack of model interpretability. Our empirical findings suggest that explainable AI models can effectively advance our understanding and interpretation of credit risks in peer to peer lending.

Future research may involve further experimentation and the application to other case studies.

## Data Availability Statement

The datasets generated for this study are available on request to the corresponding author.

## Author Contributions

All authors listed have made a substantial, direct and intellectual contribution to the work, and approved it for publication.

## Conflict of Interest

NB, DM, and JP are employed by the company FIRAMIS. The remaining author declares that the research was conducted in the absence of any commercial or financial relationships that could be construed as a potential conflict of interest.
